# QCard-NM: Developing a semiautomatic segmentation method for quantitative analysis of the right ventricle in non-gated myocardial perfusion SPECT imaging

**DOI:** 10.1186/s40658-023-00539-6

**Published:** 2023-03-23

**Authors:** Seyed Mohammad Entezarmahdi, Reza Faghihi, Mehran Yazdi, Negar Shahamiri, Parham Geramifar, Mahdi Haghighatafshar

**Affiliations:** 1 Nuclear Engineering Department, Shiraz University, Shiraz, Iran; 2 Nuclear Medicine and Molecular Imaging Research Center, Namazi Teaching Hospital, Shiraz University of Medical Sciences, Shiraz, Iran; 3 School of Electrical and Computer Engineering, Shiraz University, Shiraz, Iran; 4 Department of Computer Science and Engineering and IT, Shiraz University, Shiraz, Iran; 5 Research Center for Nuclear Medicine, Tehran University of Medical Sciences, Tehran, Iran; 6 Department of Nuclear Medicine, School of Medicine, Shiraz University of Medical Sciences, Shiraz, Iran

**Keywords:** MPI SPECT, Right ventricle, Segmentation, Quantitative analysis

## Abstract

**Background:**

Recent studies have shown that the right ventricular (RV) quantitative analysis in myocardial perfusion imaging (MPI) SPECT can be beneficial in the diagnosis of many cardiopulmonary diseases. This study proposes a new algorithm for right ventricular 3D segmentation and quantification.

**Methods:**

The proposed Quantitative Cardiac analysis in Nuclear Medicine imaging (QCard-NM) algorithm provides RV myocardial surface estimation and creates myocardial contour using an iterative 3D model fitting method. The founded contour is then used for quantitative RV analysis. The proposed method was assessed using various patient datasets and digital phantoms. First, the physician’s manually drawn contours were compared to the QCard-NM RV segmentation using the Dice similarity coefficient (DSC). Second, using repeated MPI scans, the QCard-NM’s repeatability was evaluated and compared with the QPS (quantitative perfusion SPECT) algorithm. Third, the bias of the calculated RV cavity volume was analyzed using 31 digital phantoms using the QCard-NM and QPS algorithms. Fourth, the ability of QCard-NM analysis to diagnose coronary artery diseases was assessed in 60 patients referred for both MPI and coronary angiography.

**Results:**

The average DSC value was 0.83 in the first dataset. In the second dataset, the coefficient of repeatability of the calculated RV volume between two repeated scans was 13.57 and 43.41 ml for the QCard-NM and QPS, respectively. In the phantom study, the mean absolute percentage errors for the calculated cavity volume were 22.6% and 42.2% for the QCard-NM and QPS, respectively. RV quantitative analysis using QCard-NM in detecting patients with severe left coronary system stenosis and/or three-vessel diseases achieved a fair performance with the area under the ROC curve of 0.77.

**Conclusion:**

A novel model-based iterative method for RV segmentation task in non-gated MPI SPECT is proposed. The precision, accuracy, and consistency of the proposed method are demonstrated by various validation techniques. We believe this preliminary study could lead to developing a framework for improving the diagnosis of cardiopulmonary diseases using RV quantitative analysis in MPI SPECT.

**Supplementary Information:**

The online version contains supplementary material available at 10.1186/s40658-023-00539-6.

## Introduction

Myocardial perfusion imaging (MPI) is a powerful diagnostic and prognostic method in patients with known or suspected coronary artery disease and ventricular function [1]. MPI has been widely utilized in clinical practice for more than 4 decades. There is a large amount of research supporting its diagnostic performance, utility in prognostication, and risk stratification. The American and European guidelines have emphasized the role of radionuclide imaging in patients with known or suspected coronary artery disease (CAD) [2, 3]. The main interest of MPI is focused consistently on left ventricular (LV) perfusion, while the lack of right ventricular (RV) uptake limits RV assessment in MPI diagnosis.

The following four clinical factors may result in an elevated stress RV-to-LV (RV/LV) uptake ratio: increased myocardial blood flow within RV, a global reduction in LV tracer uptake, RV hypertrophy, and RV dilation [4–6]. However, in two latter cases, an abnormally increased RV tracer uptake occurs in both rest and stress images.

Some works have demonstrated that assessing RV perfusion, function, and metabolism in patients with cardiovascular diseases can offer valuable diagnostic and prognostic information [7–11]. Additionally, it has been demonstrated that patients with incident RV abnormalities on low-risk SPECT (single-photon emission computerized tomography) imaging studies may be more susceptible to adverse clinical outcomes [12]. Studies reveal that quantification analysis of the RV in MPI SPECT may improve the diagnosis of RV abnormalities [13–17]. As an example, it has been shown that RV function is a key factor, which along with pulmonary artery hemodynamic variables can improve the accuracy of the prognostic stratification in patients with heart failure [18]. Furthermore, in heart failure with preserved ejection fraction (HFpEF), both RV dysfunction and pulmonary hypertension (PH) are highly prevalent [19]. As detected by the MPI scan, RV ischemia and hemodynamic abnormalities indicating RV dysfunction are directly correlated in patients with primary PH [20]. Moreover, some papers have reported that increased RV-to-LV uptake ratio with stress is associated with severe CAD, especially in the absence of severe proximal right CAD [5, 6, 21, 22]. Another study has shown that RV reversibility is seen in patients with LV inferior ischemia, and an automatic quantitative package can track it. They suggested additional research to determine the diagnostic value of quantitative RV analysis on MPI [23].

However, RV abnormality detection is still a challenging issue. For instance, a change in the sensitivity and resolution of the gamma camera may alter the count statistic of the RV region. Additionally, the RV measured counts may be impacted by attenuation correction, resolution modeling, and image acquisition and reconstruction techniques [24]. Furthermore, visual evaluation to identify RV abnormalities is prone to misinterpretation and uncertainty in the presence of LV hypertrophy or a general decrease in LV count [24]. Moreover, RV strain and extracardiac activity can differ between pharmacological and exercise stress protocols [8]. Extracardiac activity, mainly hepatic activity, may affect correct RV volume and perfusion measurement. Therefore, a precise quantitative analysis of RV uptake, size, and function is conducive to characterize RV abnormality more consistently [24].

Several commercial and non-commercial packages have been developed for LV myocardial quantitative analysis in MPI. Some researchers used geometrical models to segment the LV in perfusion images [25–29]. For example, Cedars-Sinai Quantitative Perfusion SPECT software (QPS) segments and quantifies the LV based on an ellipsoidal model [29]. In another study, researchers used a 3D heart shape model and the active shape algorithm for LV segmentation [28]. Another approach accomplished LV segmentation based on Dijkstra’s algorithm [30]. Supervised deep learning methods have also been developed for LV segmentation [31, 32].

Although the RV segmentation and analysis was developed and advanced in cardiac blood pool SPECT [33–37], RV automatic segmentation in MPI SPECT has been less developed. Since RV myocardium thickness and blood flow are less than the LV, it is challenging to perceive the RV myocardium in normal subjects [38]. Recently, Cedars-Sinai QPS software has developed an automatic RV segmentation which presents just RV functional parameters that, based on our observation, is not so robust in case of the intense extracardiac activity adjacent to the RV position.

As accurate segmentation of the RV is an essential step for MPI quantitative analysis, a novel technique for RV segmentation, named QCard-NM (Quantitative Cardiac analysis in Nuclear Medicine imaging), is proposed in this study. In the proposed method, the LV is segmented first using an improved technique based on the Cedars-Sinai approach [39]. An initial model for the RV is considered based on the position and shape of the LV. Then, the RV mid-myocardial points for each normal profile extracted from the model are found. Using these points, the model would be updated iteratively until the arbitrary condition is satisfied. The mid-myocardial surface and the myocardial contour are extracted using the final model. In addition, the quantification parameters and polar map are extracted from the segmented RV. These quantitative parameters have been shown to be valuable in CAD detection.

## Material and methods

### Segmentation algorithm

In our proposed approach, we named it QCard-NM, the LV is segmented first, and RV segmentation starts based on the position of the LV. Based on the RV shape in the MPI scan, an appropriate model is generated for RV segmentation, as discussed later in this section. Epicardial and endocardial surfaces are then determined via an iterative process based on the fitted model. The segmented RV volume is then used for quantification.

### LV segmentation

The transversal reconstructed image is rotated based on the predefined constant angle to estimate the short-axis view. To calculate the predefined rotating angle, a group of images was automatically rotated into short-axis slices by the method described in [25]. Then, the average angle of the LV long axis of these images was considered the prior rotation angle. The image is binarized by a threshold of 50% of the voxels’ maximum count value on the upper-right side of the transversal slices. Based on the location and size of the clusters, one is selected as the LV initial cluster. The bounding box of the initial cluster is drawn and can be confirmed or changed by the user. Radial profiles (10 longitudinally and 10 latitudinally) are generated from the center of the box in 3D short-axis slices. The count profiles are averaged and its maximum is suggested as the intra-patient threshold [39]. The threshold is used to obtain the final LV cluster. An initial ellipsoidal model is fitted to the selected cluster. Radial count profiles originating from the ellipsoid center are extracted. The best point on each count profile that depicts where the epicardial and endocardial surfaces are located is chosen. A new ellipsoidal model is fitted to these selected points. These steps are iterated until an arbitrary objective is attained. The image volume is rotated based on the longest radius of the final ellipsoidal model to adjust LV orientation in the short-axis view. Finally, the valve plane position is determined based on the mid-myocardial count distribution. The flowchart of LV segmentation algorithm is provided in Additional file 1: Flowchart S.1. More comprehensive details for LV segmentation can be found in [29].

### RV segmentation

#### RV geometrical model

**Fig. 1:**
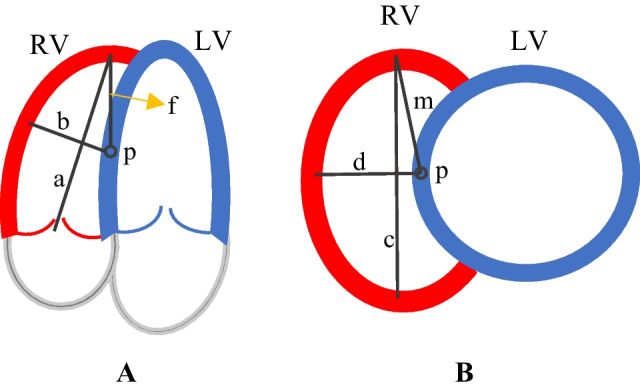
Left (blue) and right (red) ventricles schema—**A** horizontal long-axis view—**B** short-axis view

A semiautomatic model-based algorithm is used to segment the RV. Segmentation starts by assuming an initial spherical model centered on the septum wall. It can be proved that the spherical model is suited to the RV geometry, as follows. Figure 1 shows the short- and long-axis slices of the ventricles. “*P*” is denoted as the sphere center. The distance between *P* and the apex is called “*f*” which is equal to Eq. (1).

1 $$f = \frac{1}{2}\sqrt {a^{2} + b^{2} }$$

RV sphericity index is defined by Eq. (2) where “*a*” and “*b*” are the short and long axis of the LV, respectively (see Fig. 1A).

2 $$x = \frac{a}{b}$$

If the spherical model is suitable, “*f*” and “*b*” should be equal. Based on the population reported in [40], the average end-systolic and end-diastolic sphericity indices are equal to 2 and 1.7, respectively. Consequently, according to Eq. (3), the value of $\frac{f}{b}$ is 1.11 and 0.98 for the end systolic and end diastolic, respectively. “*b*” and “*f*” are close together; thus, a sphere can be an acceptable model.

3 $$\frac{f}{b} = \frac{1}{2} \sqrt {\left( \frac{a}{b} \right)^{2} + 1} = \frac{1}{2}\sqrt {x^{2} + 1}$$

Another parameter for analyzing the RV geometry is the eccentricity index that is calculated as a ratio of “*c*”–“*d*” where “*c*” and “*d*” are the longest and shortest diameter of the short-axis view of the mid-part RV (see Fig. 1B). Assuming “*P*” as the sphere center, “*m*” should be close to “*d*” in Fig. 1B. Assume m is close to $\frac{c}{2}$. Therefore, $\frac{m}{d}$ is calculated by Eq. (4).

4 $$\frac{m}{d} \approx \frac{\frac{c}{2}}{d} = \frac{1}{2}\frac{c}{d} = \frac{1}{2} {\text{eccentricity}}\;{\text{ index}}$$

Again, based on the normal range of eccentricity index reported in [40], the average $\frac{m}{d}$ is equal to 0.9 and 1 for the end systolic and end diastolic, respectively. It indicates that in the short-axis view, the longest and shortest radii of the mid-part RV surface model are almost identical. Thus, the spherical model is suited to non-gated MPI SPECT.

To reach the initial sphere center, the LV center point is shifted by the length of the LV ellipsoid’s smallest radius on the *X*-axis and one-fifth of the ellipsoid’s longest radius on the *Z*-axis. The preferred sphere radius is assumed to be 1.5 times greater than the smallest radius of the LV ellipsoid model. The center and radius are chosen empirically and can be modified by the user in case of model misplacement.

The LV is excluded from the short-axis image volume. Radial RV count profiles (48 longitudinally and 96 latitudinally) originating from the sphere center are extracted from the image volume. The local maxima of each profile are found. The local maximum that satisfies the following conditions is chosen: 1—Its distance to the sphere surface is less than or equal to a pixel size (6.4 mm), and 2—the voxel count value is more than one-quarter of the maximum LV count value. The corresponding voxel count value called *C*_profile_ is used to determine the surface points. It should be mentioned that about 2000 of the total 4608 profiles satisfy these conditions. For those profiles, epicardial and endocardial surface points are chosen based on a threshold calculated by Eq. (5).

5 $${\text{Threshold}} = \frac{0.8}{{1 - 0.4\left( {C_{\max } - C_{{{\text{profile}}}} } \right)}}C_{{{\text{profile}}}}$$

where *C*_max_ is equal to 50% of the maximum LV count value, voxels above the threshold are assumed as RV myocardium per profile, and the outer and the inner parts of the myocardium are epicardial and endocardial surfaces, respectively. Delineating epicardial and endocardial surfaces depends on the *C*_max_ and *C*_profile_ values differences, as well as *C*_profile_ value, as shown in Eq. (5). Following this thresholding step, the distance between each surface point and the sphere is measured along each profile. Those endocardial and epicardial surface points whose distances are more than two pixels size (12.8 mm) are considered far-off points. The far-off points do not contribute in the proceeding steps. Along each profile, the mid-myocardial surface point is defined in the middle of the epicardial and endocardial points. The new sphere is fitted to these mid-myocardial points by the least square method. This is repeated until sphere equation coefficients reach convergence. This iterative model fitting is necessary to preserve the repeatability of the algorithm. The final model is the best sphere fitted to the mid-myocardial surface.

#### Epicardial and endocardial surface points

The final sphere model delineates the epicardial and endocardial surface points. Radial profiles (120 longitudinally and 320 latitudinally) originating from the sphere center are extracted from the image volume. Epicardial, endocardial, and mid-myocardial surface points are delineated as described previously. A new sphere is fitted to the mid-myocardial points. Each selected epicardial and endocardial surface point which does not meet the local maxima criterion is replaced with a point from the spherical model.

The RV valve plane is determined along the LV valve plane in the vertical long-axis view and parallel to the *X*-axis in the horizontal long-axis view. Voxels enclosed by the endocardial surface and the valve plane are used to determine RV cavity volume. The intersection of the RV and LV valve planes is placed on the LV epicardial surface. A fifth-degree polynomial equation (with boundary condition) is used to construct the mid-myocardial surface. Polynomial is fitted to the surface points by the Levenberg–Marquardt method [41]. RV myocardium is determined by dilating the mid-myocardial surface. Epicardial and endocardial surfaces are the outer and inner surfaces of the myocardium, respectively. The surfaces are delimited by the valve plane.

**Fig. 2:**
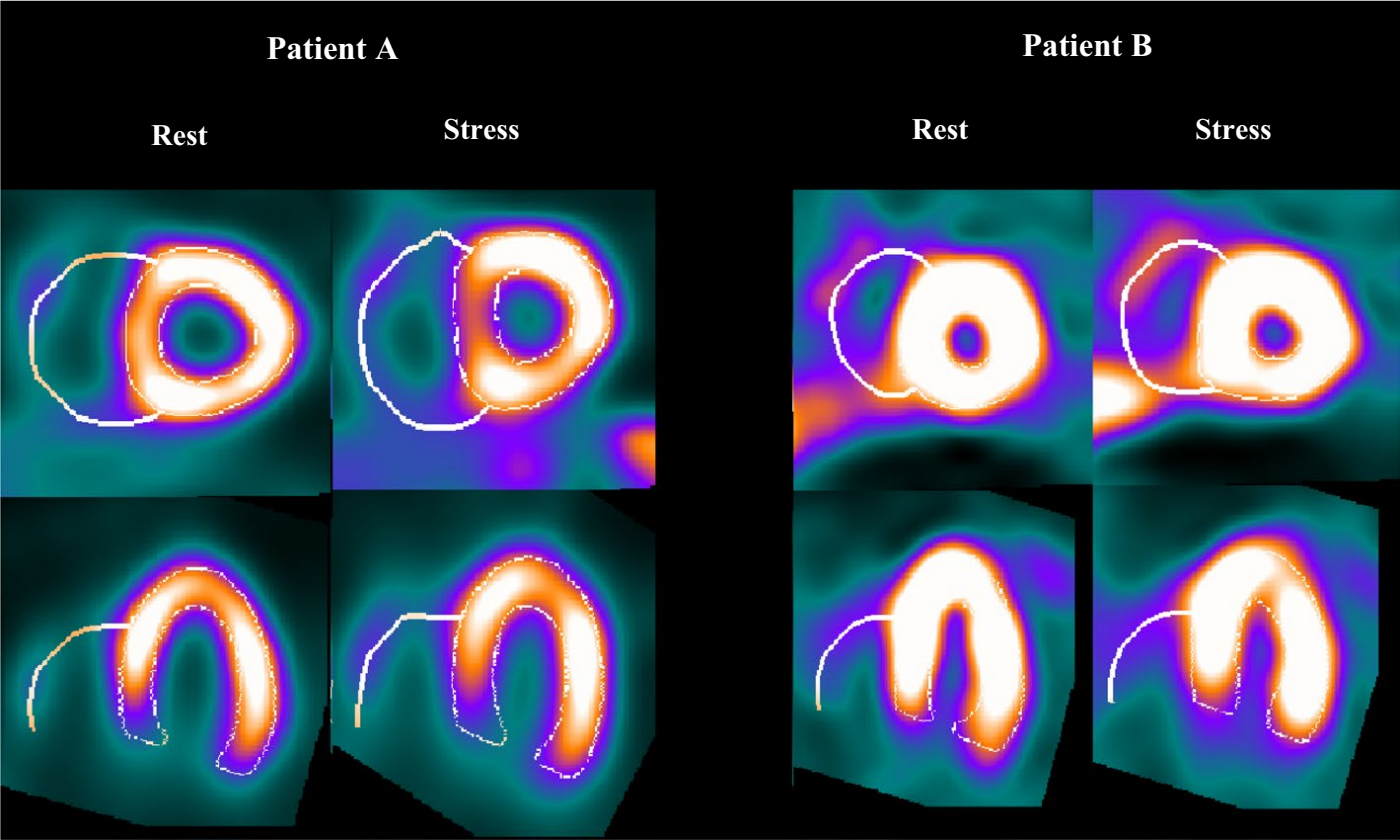
Two sample patients’ right and left ventricular segmentation by QCard-NM. The proposed algorithm can detect the RV even in the presence of intense extracardiac activity or when the RV is partially visible, as seen in the figure

**Fig. 3:**
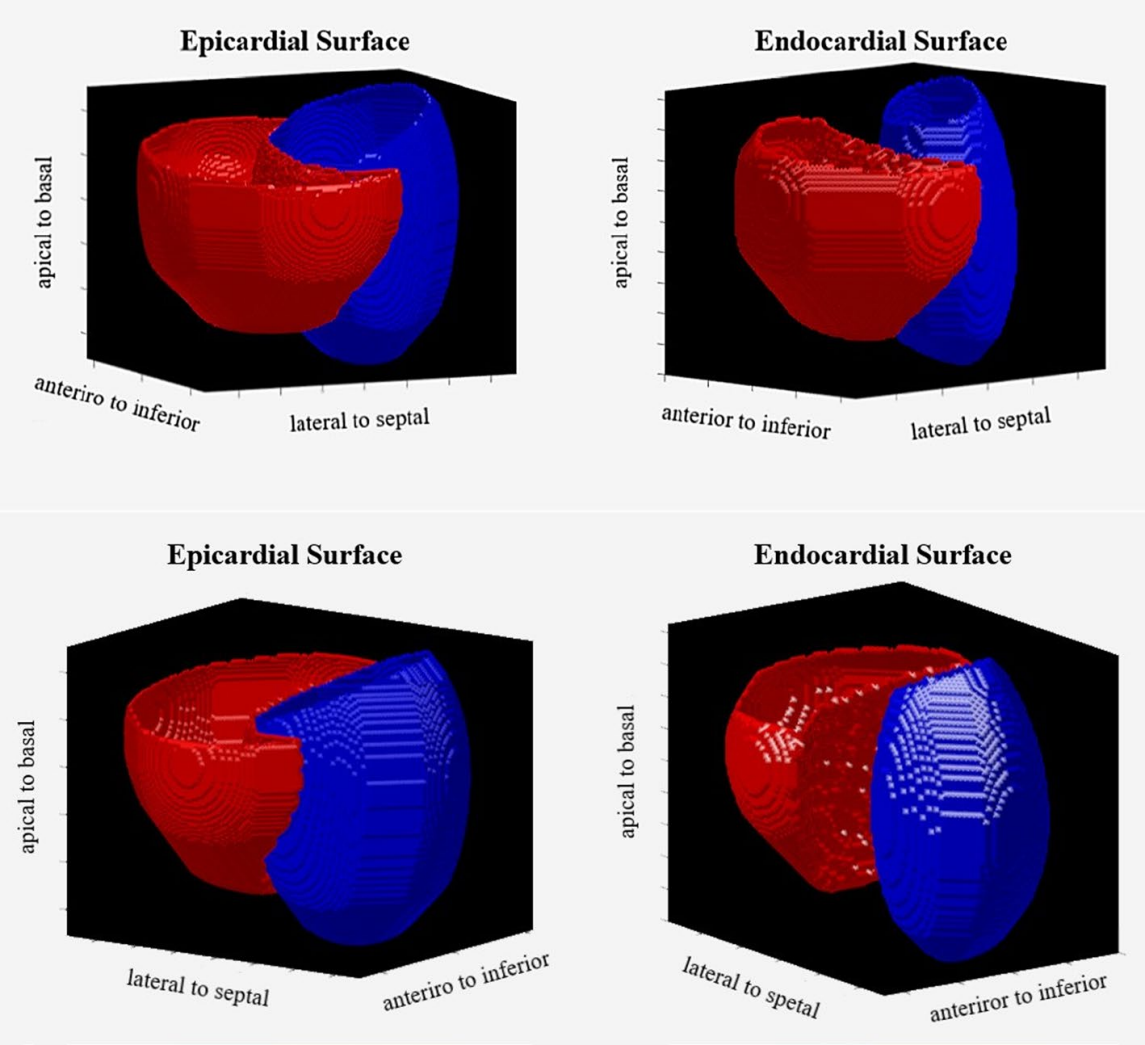
A sample of the 3D surface rendering image. Left ventricular (blue) and right ventricular (red) are segmented. Epicardial and endocardial surfaces are illustrated from two different view angles (top and bottom rows)

Since the normal RV wall thickness is less than one voxel size (where the system resolution is 9 mm and the voxel size is 6.4 mm) in an MPI scan, the apparent RV myocardium thickness is due to a partial volume effect. As a result, the mid-myocardium surface is the best estimator for the correct RV wall position. The final visual result of the segmented RV is the 3D mid-myocardium surface. Figure 2 shows the segmented LV and RV contours in 2D slice view for two sample patients. Figure 3 shows a sample of 3D surface rendering of a segmented cardiac volume. Epicardial and endocardial surfaces from two different view angles are illustrated in Fig. 3. Additionally, the flowchart of the RV segmentation algorithm is presented in Additional file 1: Flowchart S.2.

#### RV polar map and quantification

**Fig. 4:**
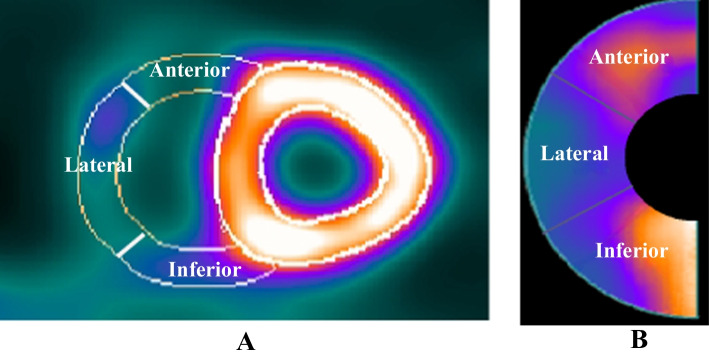
Representation of three subsegments of the RV wall in the **A** short-axis view and **B** the polar map; The RV wall is segmented into three subsegments: anterior, lateral, and inferior

**Fig. 5:**
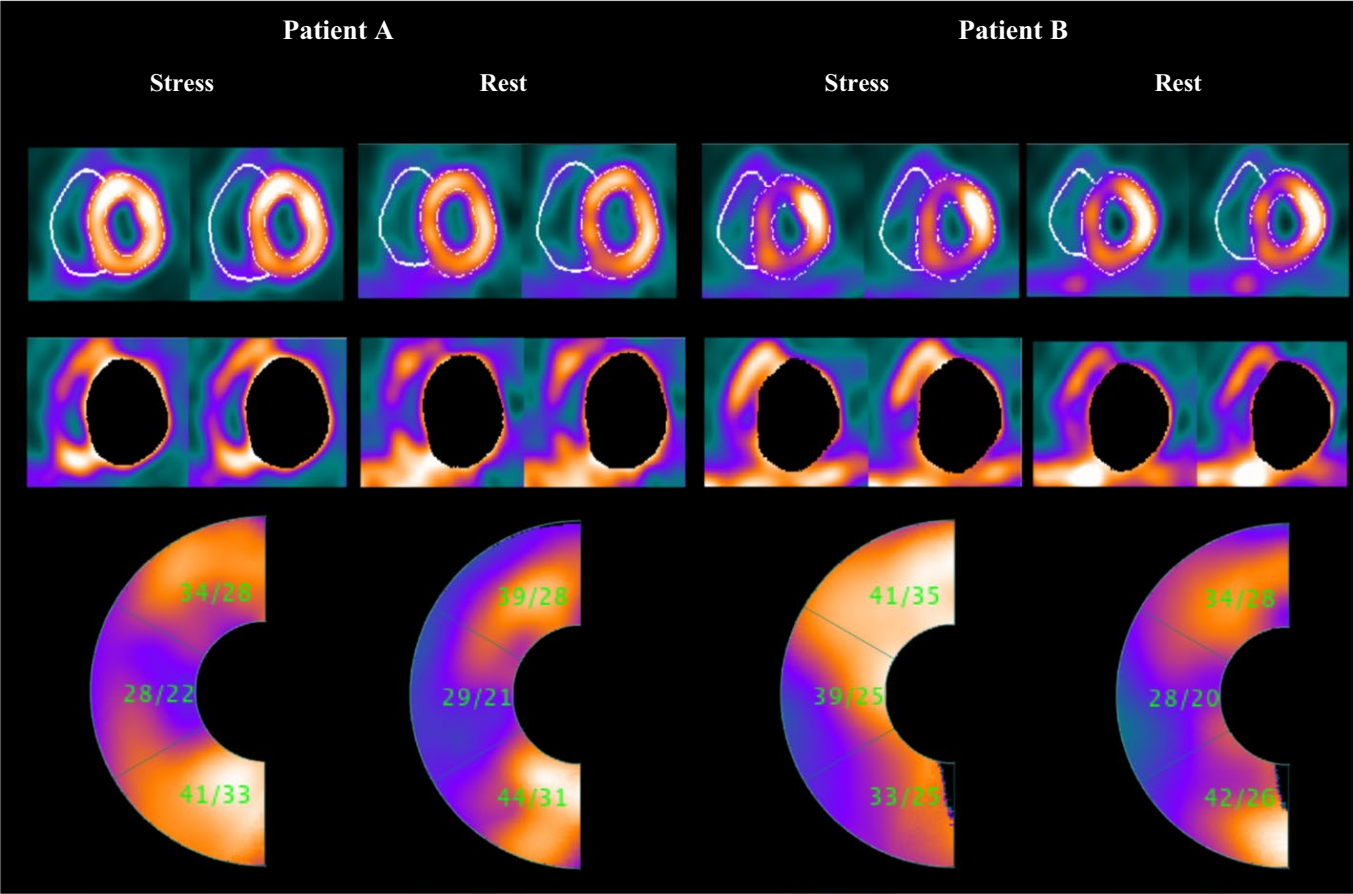
The stress/rest scan for two sample patients. Patient **A** a normal individual with no significant CAD based on CA report. Patient **B** a 3VD patient based on CA report. For each individual, three rows of images are represented. First row: the short-axis image slices are represented. Second row: LV is extracted automatically by the proposed segmentation algorithm to enhance the RV visualization. Third row: the RV quantified polar map by proposed method. (The numerical values represent the maximal/average RV/LV uptake ratio)

The maximum count value is chosen in each myocardium sector to generate the polar map. Each sector is a part of the myocardium volume at a specific angle from the center of the RV spherical model. RV polar map generation is the same as that previously used for the LV polar maps [42]. Figure 4 shows how the RV polar map is divided into three segments. For quantitative analysis, both the maximum and the average of the counts can be computed for each segment. RV measured count is commonly normalized to the maximum LV count. Figure 5 shows two examples of quantified polar maps demonstrating the maximal/average RV/LV uptake rate in each segment.

### Patient population

This study enrolled four retrospective patient populations (124 patients, 50% men). All the patients underwent Tc-99 m sestamibi MPI SPECT examination. The first population includes 24 individuals who underwent two-day rest/stress SPECT/CT MPI protocols in which CT scans were acquired in both the rest and stress phases. In this paper, we call this population as SPECT/CT dataset.

The second population consists of 20 patients who underwent prone position MPI examination immediately after the supine imaging. Prone position images were acquired at the stress phase to lessen inferior wall attenuation. This dataset is called a supine/prone dataset in this literature.

Sixty patients referred for both stress/rest MPI and coronary angiography (CA) were enrolled in the third dataset, named SPECT/Angio dataset in the text. The CA was examined within 2 months after the MPI scan. Based on the CA examination and the clinical reports, patients who were right dominant and belonged to one of the following four subgroups were enrolled in this dataset:

1. *Normal* patients with no significant CAD.
2. *RCA > LAD, LCX* patients with proximal or mid-RCA significant stenosis with no significant lesion in the left coronary system. (Significant stenosis: > 50%)
3. *3VD* Patients with severe three-vessel diseases.
4. *LAD, LCX > RCA* patients with proximal or mid-LAD or LCX significant stenosis with no significant lesion in RCA. (Significant stenosis: > 50%)

Fourth dataset, called as normalcy group in this paper, consists of 20 individuals who were deemed to be at low probability of having CAD based on the following criteria:

1. *None of the following coronary risk factors* hypertension, hyperlipidemia, smoking, diabetes mellitus, and chronic kidney disease (CKD).
2. No stress-induced symptoms with successful complete stress protocol.
3. No evidence of fixed or reversible perfusion defect on rest/stress MPI SPECT.

Patients’ clinical data, including demographic information, risk factors, MPI, and CA results, were collected from the electronic medical records and are presented in Table 1.

**Table 1:** Clinical characteristics, MPI data, and angiographic results of the patient populations

Characteristics	SPECT/CT dataset	Supine/Prone dataset	SPECT/Angio dataset	Normalcy dataset
Number	24	20	60	20
Age (years)	56.5 ± 8.6 (41–69)	60.9 ± 10.9 (37–83)	61.9 ± 10.1 (36–90)	56.7 ± 11.5 (39–80)
Male sex	9 (37%)	14 (70%)	32 (53%)	7 (35%)
*Risk factors*				
Obesity	5 (21%)	4 (20%)	18 (30%)	3 (15%)
Diabetes mellitus	7 (29%)	4 (20%)	21 (35%)	0
Hypertension	13 (54%)	9 (45%)	44 (73%)	0
Hyperlipidemia	8 (33%)	6 (30%)	23 (38%)	0
CKD	1 (4%)	0 (0%)	1 (2%)	0
Current smoker	5 (21%)	3 (15%)	16 (27%)	0
Family history	3 (12%)	2 (10%)	15 (25%)	4 (20%)
*Stress protocol*				
Dipyridamole stress protocol	24 (100%)	20 (100%)	58 (97%)	20 (100%)
Dobutamine stress protocol	0	0	2 (2%)	0
Maximum heart rate	88.2 ± 10.2 (71–103)	84.7 ± 15.2 (62–115)	94.5 ± 14.4 (65–133)	99.25 ± 15.3 (82–131)
*Interpretation of MPI throughout the LV myocardium*				
Negative for appreciable ischemia	7 (29%)	13 (65%)	24 (30%)	20 (100%)
Mild-to-moderate ischemia	11 (46%)	6 (30%)	25 (31%)	0
Severe ischemia	6 (25%)	1 (5%)	28 (35%)	0
MI; MI + ischemia	0	0	3; 3 (7%)	0
Normal function	21 (87%)	18 (90%)	46 (57%)	0
Stress EDV (ml)	84.3 ± 21.6 (28–215)	105 ± 36.9 (48–204)	78.1 ± 38.5 (22–224)	76.2 ± 13.14 (62–99)
Stress ESV (ml)	34.1 ± 8.1 (9–48)	37.7 ± 21.8 (6–90)	32.7 ± 26.2 (6–138)	24.25 ± 7.49 (13–73)
Stress EF (ml)	63.2 ± 4.9 (23–85)	65.4 ± 8.9 (46–75)	56.9 ± 23.0 (4–85)	68.6 ± 6.2 (61–79)
*Angiographic results (N* = *60)*				
Normal	–	–	7 (12%)	–
RCA > LAD, LCX	–	–	11 (18%)	–
3VD	–	–	17 (28%)	–
LAD, LCX > RCA	–	–	25 (42%)	–

Values are presented as mean ± SD (range) or N (%) as appropriate

*CKD* Chronic kidney disease, *EDV* End-diastolic volume, *ESV* End-systolic volume, *EF* Ejection fraction, *LV* Left ventricular, *MPI* Myocardial perfusion imaging, *MI* Myocardial infarction, *LAD* Left ascending coronary, *LCX* Left circumference coronary, *RCA* Right coronary artery, *3VD* Three-vessel diseases

### Digital phantoms

The non-uniform rational B-spline (NURB) extended cardiac-torso phantoms were generated in the XCAT package version 2.0 [43]. Thirty-one phantoms (16 adult males and 15 adult females) were generated with various heart sizes and abnormalities to simulate non-gated SPECT scans. (The RV cavity volume ranged from 40 to 220 ml, and defects were located on the basal and apical RV wall with three different severity levels.) The XCAT software also provided the truth values of all chambers’ volumes.

The Monte Carlo simulation program, SIMIND version 6.2.1, simulated summed myocardial perfusion images based on the XCAT phantoms and the corresponding attenuation maps [44]. Parameters of the SIMIND software were set to model the Siemens Symbia T2 hybrid SPECT/CT gamma camera (Symbia T2) (Siemens Medical Solutions Inc., Hoffman Estates, IL., USA). The Imaging protocol was set the same as the one we used in the clinic.

### SPECT acquisition and processing protocol

A two-day Tc-99 m sestamibi protocol was used to perform the rest/stress scan. In both the rest and stress phases, the administered doses were based on the weight of the patients (8 MBq/kg) [45]. Rest acquisition started 60 min after the tracer was injected. Pharmacological stress was induced by an infusion of dipyridamole or dobutamine. Three minutes after the dipyridamole slow infusion, the radiotracer was injected. Stress acquisition started 45–90 min after the injection.

The SPECT studies were acquired with a dual-head detector camera, with low-energy, high-resolution collimators, a 20% symmetrical window at 140 keV, and a 64 × 64 matrix size. For each scan, 32 projections with 28 s per projection were acquired. Images were reconstructed using OSEM iterative method (four iterations and four subsets [46]), and the Butterworth post-reconstruction filter with order = 5 and cutoff = 0.5 was applied to smooth the images by QPS software v2015.1.

Images in SPECT/CT dataset were acquired by utilizing Siemens Symbia T2 dual-headed SPECT/CT gamma camera. CorCam Gamma Camera System (DDD-Diagnostic, Denmark) was used to acquire the images of patients in three other datasets. No attenuation, scatter, and detector response corrections were applied to maintain the generality of the proposed approach.

### Evaluation strategies

Four strategies were performed in this paper to evaluate the performance of the proposed RV segmentation algorithm.

1. *Spatial similarity assessment* An experienced nuclear medicine physician analyzed two short-axis and two horizontal long-axis views for each patient in the SPECT/CT dataset. The corresponding CT images of the selected views were also fused to the SPECT images to improve certainty. The RV contour drawn manually by the physician in each slice was compared to the contour determined by QCard-NM algorithm.
2. *Repeatability assessment* The supine/prone dataset was used to assess the repeatability of the proposed algorithm and to compare it with the QPS algorithm. The QCard-NM and QPS algorithms were applied to the dataset images, and the RV cavity volumes were calculated for both the supine and prone stress images. In an ideal situation, the RV cavity volume calculated on supine and prone imaging would be the same. Considering this, the repeatability of QCard-NM and QPS was assessed in this study. Additionally, in this dataset, the RV cavity volume measured by the QCard-NM and QPS algorithms was compared to each other.
3. *Digital phantom assessment* Phantom studies were utilized to quantify the accuracy of the proposed algorithm’s output. The XCAT phantoms were fed into the SIMIND software, which simulated the non-gated SPECT. The SIMIND outputs were reconstructed, and transversal images were segmented using the QCard-NM and QPS algorithms. The calculated RV cavity sizes were compared to their actual sizes (originating from the XCAT phantom).
4. *RV/LV uptake ratio assessment* RV-to-LV uptake ratio (RV/LV) is defined as the RV pixel count divided by the maximum LV count. It has been demonstrated that CAD is related to the maximal RV/LV uptake ratio [5, 6, 21]. The RV/LV uptake ratio was manually generated in the previous articles from the RV lateral segment (free wall). In the fourth assessment, we investigated whether it is possible to classify patients with CAD by utilizing the proposed semiautomatic RV segmentation and quantification approach. The third and fourth datasets were used in this part. Initially, to determine the correctness of RV/LV quantitation, an expert physician visually identified those patients with high RV/LV uptake ratio. Quantitative values obtained from QCard-NM were compared to the physician’s visualized categorization. Then, a study similar to [5] was conducted to investigate the capability of semiautomatic RV quantitative analysis for classifying those patients with CAD (with no significant stenosis in RCA)

### Statistical analysis

All statistical calculations were performed by use of MedCalc statistical software Version 20.104 (MedCalc, Mariakerke, Belgium). A *P* value less than 0.05 was considered significant.

Physicians’ manually drawn contours of the RV myocardium were compared with QCard-NM's determined contours using the Dice similarity coefficient (CSD) [47].

Mean absolute percentage error (MAPE) was utilized to show the difference between the RV volumes measured in prone and supine scans.

6 $${\text{MAPE}} = \frac{100}{N}\sum \left| {\frac{{\left( {x_{{\text{s}}} - x_{{\text{p}}} } \right)}}{{\frac{{\left( {x_{{\text{s}}} + x_{{\text{p}}} } \right)}}{2} }}} \right|$$

where $x_{{\text{s}}}$ and $x_{{\text{p}}}$ are the measured RV volumes in supine and prone scanning, respectively.

Besides, the coefficient of repeatability (CR) was calculated as 2.77 times the within-subject standard deviation. Bland–Altman analysis was utilized to study the pairwise percentage RV volume difference between prone and supine scanning [48]. Means, as well as 95% limits of agreement (LoA), were reported. Since the Shapiro–Wilk test accepted the normal distribution hypothesis of supine and prone scanning datasets, the paired sample *t* test was applied to compare the results.

Moreover, in this dataset, the Bland–Altman graph and paired sample t test were also used to compare the measured RV volumes between the QCard-NM and QPS algorithms.

In the phantom study, the measured RV volumes were compared to the simulated actual sizes with the MAPE metric formulated as Eq. 7:

7 $${\text{MAPE}} = \frac{100}{N}\sum \left| {\frac{{\left( {x_{m} - x_{{{\text{actual}}}} } \right)}}{{x_{{{\text{actual}}}} }}} \right|$$

where $x_{m} { }$ and $x_{actual} { }$ are the measured RV volume and the phantom actual size, respectively.

Scatter plot was presented to see the relationship between the measured and the actual volumes. Linear regression analysis was performed to extract the trend line. Pearson correlation coefficient (*r*) was used to explore the relationship between the measured RV volumes and the phantom actual sizes.

The Shapiro–Wilk test confirmed the SPECT/Angio dataset follows a non-normal distribution. Therefore, in the SPECT/Angio dataset, RV/LV uptake ratio was analyzed based on the visibility of the RV wall in stress MPI SPECT using the Mann–Whitney test. RV visibility was distinguished by an expert physician. Continuous data were presented as medians with interquartile range (IQR) and compared using the nonparametric Kruskal–Wallis ANOVA. The post hoc Conover test was used to examine the between-group differences. Box-and-Whisker plots were also plotted. Receiver operating characteristic (ROC) curve analysis was performed to evaluate the diagnostic performance of RV/LV uptake ratio to distinguish those patients with 3VD or significant LAD or LCX stenosis (with no significant RCA stenosis). The area under the ROC curve (AUC), along with sensitivity, specificity, and accuracy were reported in this statistical analysis.

## Experimental results

A total of 124 patients were included in this work. Patient characteristics such as demographic data, CAD risk factors, and MPI and CA results are shown in Table 1.

### Spatial similarity assessment

**Fig. 6:**
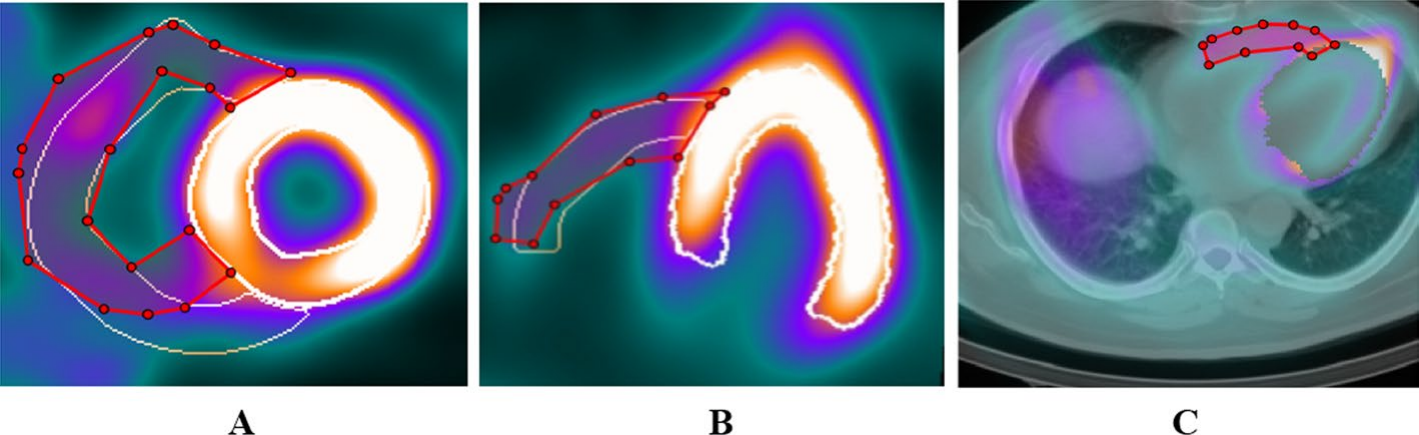
The physician’s manually drawn RV contours were compared with the proposed segmentation results. The manually drawn physician’s contour (red) and the proposed contour (white) on short-axis **A** and long-axis **B** views. **C** The fused SPECT-CT image with the physician’s contour in red

For spatial similarity assessment, the DSC value was calculated between the physician’s manually drawn RV contours and QCard-NM contours. The results were in the interval of [0.3, 0.92] with an average of 0.83 ± 0.14. The physician could delineate or modify the contours with the help of fused SPECT/CT images. The LV region in the fused SPECT/CT was masked in order to improve RV visibility. Figure 6 shows a sample of the physician’s contours in red and QCard-NM output in white. The DSC value demonstrates the effectiveness of the proposed segmentation algorithms for identifying the RV myocardium in those images that the physician was able to delineate RV contour.

### Repeatability assessment

**Fig. 7:**
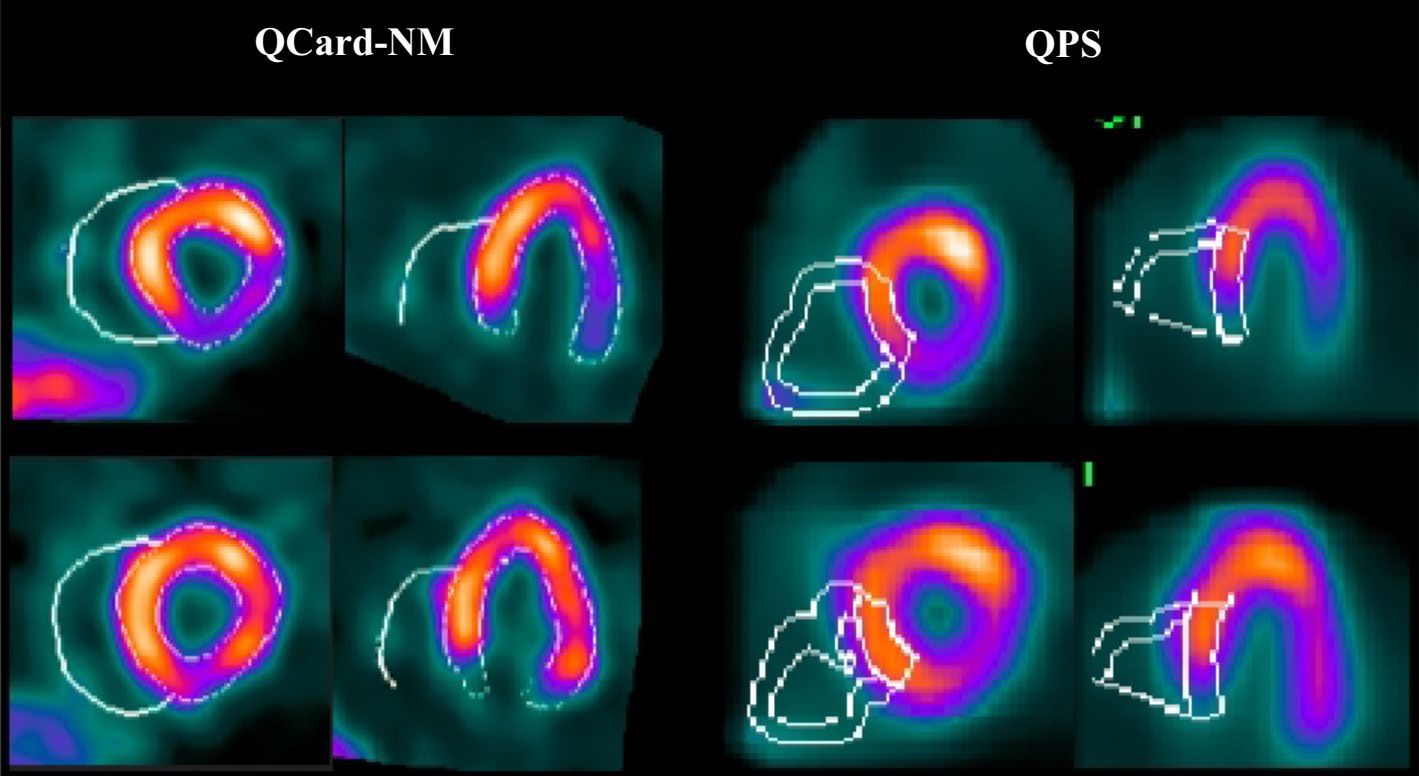
An example of repeated scans with segmentation results of the QCard-NM and QPS. First and second rows represent the supine and prone scans, respectively;

In this section, the QCard-NM and QPS algorithms were applied to the supine/prone dataset, and by using the segmented images, the LV and RV cavity volumes were measured. Results for each individual are presented in Additional file 1: Table S.1. A sample of the segmentation results from the QCard-NM and QPS algorithms in prone and supine scans is presented in Fig. 7. As can be seen, the RV contours may change between two successive scans. The average change in the RV volume size is presented in Table 2 quantitatively. The calculated MAPEs for RV cavity volume calculation between serially supine and prone scans were 12.0% and 28.0% for the QCard-NM and QPS algorithms, respectively. The MAPEs values were statistically different and show that, in this study population, the QPS error is greater than twice that of the QCard-NM algorithm. Besides, the CR was 13.57 ml for QCard-NM and 43.41 ml for the QPS. Again, a lower CR for QCard-NM indicates a lower likelihood of difference between repeated scans than the QPS.

**Table 2:** The QCard-NM and QPS algorithms repeatability assessment in the supine/prone dataset; MAPE and CR were calculated for the QCard-NM and QPS algorithms between repeated scans

Algorithm	MAPE (95% CI) (%)	CR (95% CI) (ml)
QCard-NM	12.0 (7.4–16.6)	13.57 (10.38, 19.60)
QPS	28.0 (17.0–39.1)	43.41 (33.21, 62.69)

**Fig. 8:**
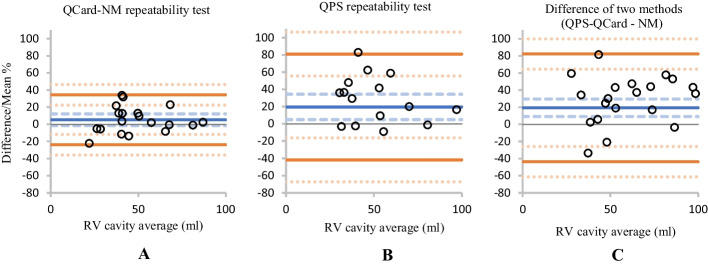
The repeatability test for the QCard-NM (**A**) and QPS (**B**) as represented using the Bland–Altman graph. Besides, the difference between QPS and QCard-NM is shown (**C**)

The Bland–Altman plot analysis was employed to investigate the calculated RV cavity volume difference between repeated scans of each algorithm. The mean of the difference, the upper and lower LoA, and their 95% confidence interval (CI) are presented in Table 3. *Y*-axis and *X*-axis in the Bland–Altman graph indicate the percentage difference between the measured cavity volumes of successive scans and the average of the measured cavity volume, respectively. The mean of the difference (blue lines), upper and lower bound of LoA (orange lines), and the corresponding 95% CI (dashed lines) were determined for the QCard-NM (see Fig. 8A) and QPS (see Fig. 8B) algorithms. The mean values extracted from the Bland–Altman analysis were 5.2% and 19.6% for QCard-NM and QPS algorithms, respectively. It demonstrates that when compared to the prone scan, QPS reveals significantly higher measured RV volume in the supine scan, whereas QCard-NM does not show a statistically significant percentage difference between supine and prone RV analysis. In comparison with QPS, QCard-NM also has a narrower level of agreement.

**Table 3:** The quantitative values of Bland–Altman graphs

	Mean (95% CI) %	LoA (Lower–Upper)	Paired samples *t* test (*P* value)
QCard-NM repeatability	5.2 (− 1.7 to 12.2)	(− 23.9 to 34.3)	0.1324
QPS repeatability	19.6 (5.0–34.3)	(− 41.8 to 81.0)	0.0002
QPS to QCard-NM difference	19.3 (9.0–29.5)	(− 43.6 to 82.2)	0.0005

Moreover, the RV cavity volumes obtained by the QCard-NM and QPS algorithms are compared in Fig. 8C. As it is shown, the RV volume measured by QPS is statistically significantly higher (about 20% larger on average) in comparison with QCard-NM.

### Digital phantom assessment

**Fig. 9:**
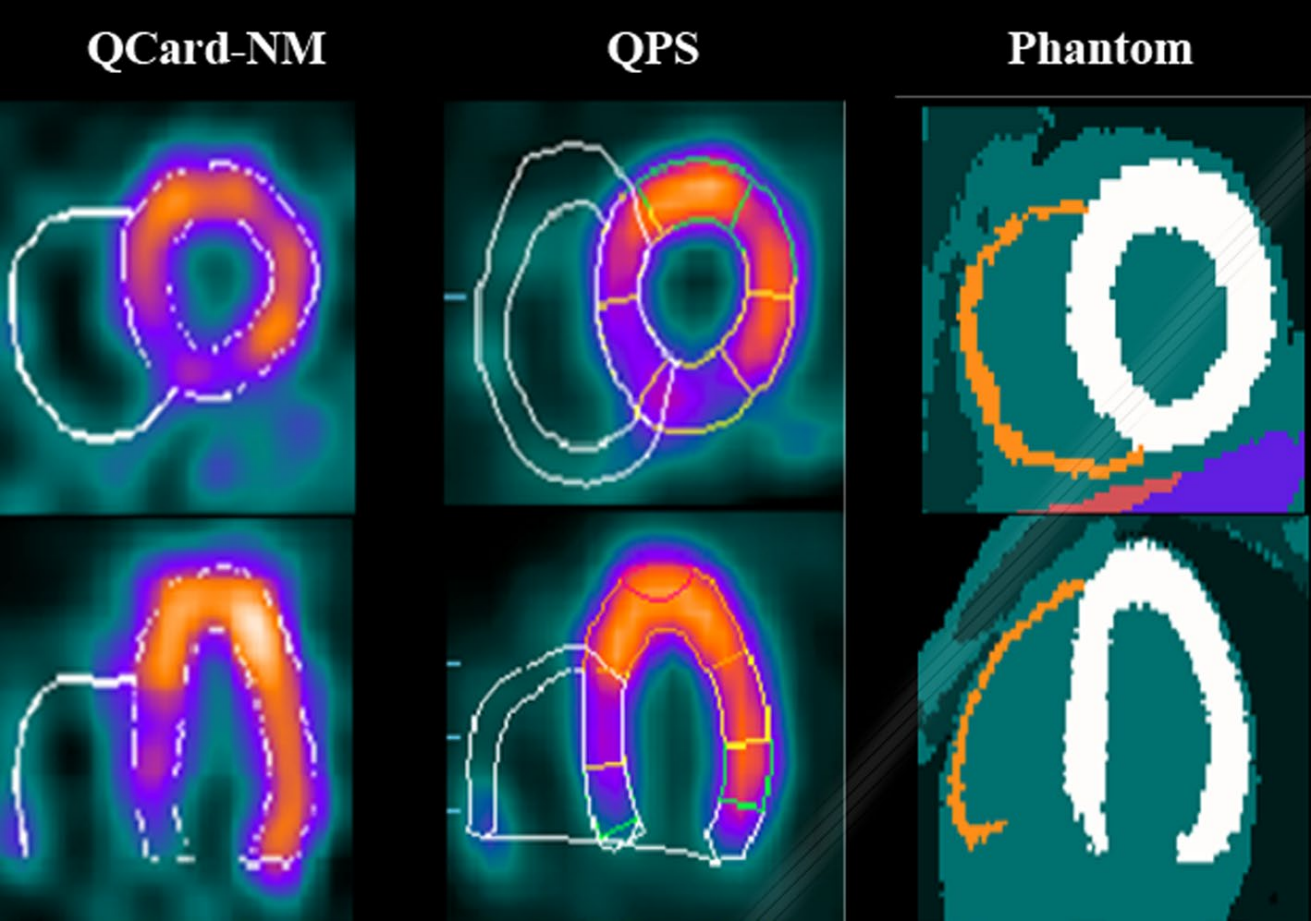
Example of simulated data with segmentation results of the QCard-NM and QPS algorithms, and the phantom image. Short axis (first row), long axis (second row)

Thirty-one XCAT phantoms with different RV sizes and uptakes were fed into the SIMIND software, which simulated the non-gated SPECT. The reconstructed images were segmented by using the QCard-NM and QPS algorithms. The difference between the measured RV cavity volumes and the actual simulated values was reported by MAPE metric. The MAPEs (95% CI) were 22.6% (15.3–29.9) and 42.2% (30.0–54.4) for the QCard-NM and QPS algorithms, respectively. An example of a segmented phantom with QCard-NM and QPS algorithms, as well as the simulated phantom, is illustrated in Fig. 9.

**Fig. 10:**
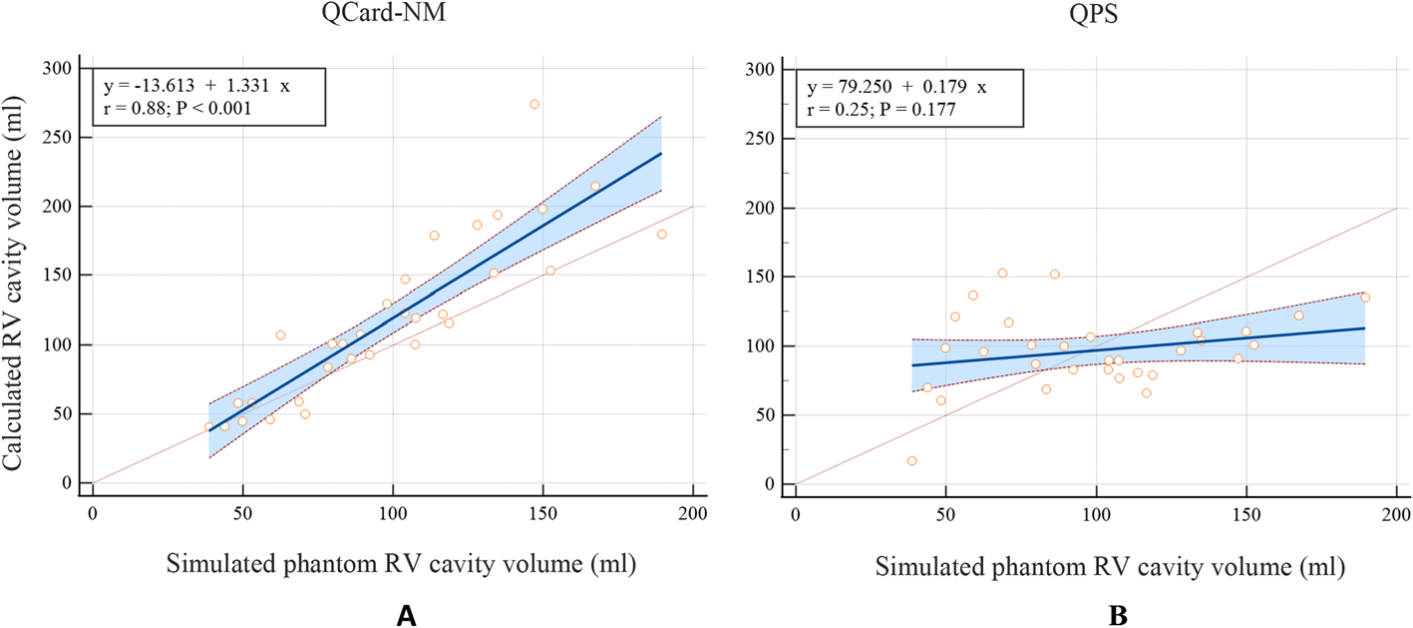
Scatter plots of the calculated volumes with **A** QCard-NM and **B** QPS versus the simulated actual RV cavity volume in phantom study. Linear regression trend line with 95% CI is exhibited in blue

Besides, a scatter plot is drawn, and the linear regression trend lines model the relationships between the measured and truth RV cavity volumes for both algorithms. The 95% CIs of the regression lines are also illustrated (see Fig. 10). The trend line belonging to QCard-NM is closer to the identity line than the QPS trend line. Thus, the proposed algorithm is more accurate in RV cavity volume calculation in digital phantoms dataset. The slope of the regression lines, the correlation coefficients, their 95% CI, and the *P* value were calculated (Table 4).

**Table 4:** Results of linear regression analysis of the calculated RV cavity volume in the QCard-NM and QPS algorithms using XCAT phantoms

Algorithms	Slope of trend Line (95% CI)	Correlation coefficient (95% CI)	*P* value
QCard-NM	1.33 (1.06–1.60)	0.88 (0.76–0.94)	< 0.001
QPS	0.18 (− 0.08 to 0.44)	0.25 (− 0.11 to 0.55)	0.177

### RV/LV uptake ratio assessment

The stress maximal RV/LV uptake ratio (within the lateral segment of the RV wall) in the SPECT/Angio and normalcy datasets was compared by the physician’s visual assessment. The maximal RV/LV was lower in individuals with imperceptible stress RV uptake (median (IQR): 28 (23–32.5)) than in patients with visible stress RV (median (IQR): 36.5 (31.5–41.5)). This difference was statistically significant (*P* value: 0.0019). Therefore, it can be concluded that the quantitative RV/LV uptake ratio in a stress MPI scan appears to be statistically associated with RV visual assessment.

**Fig. 11:**
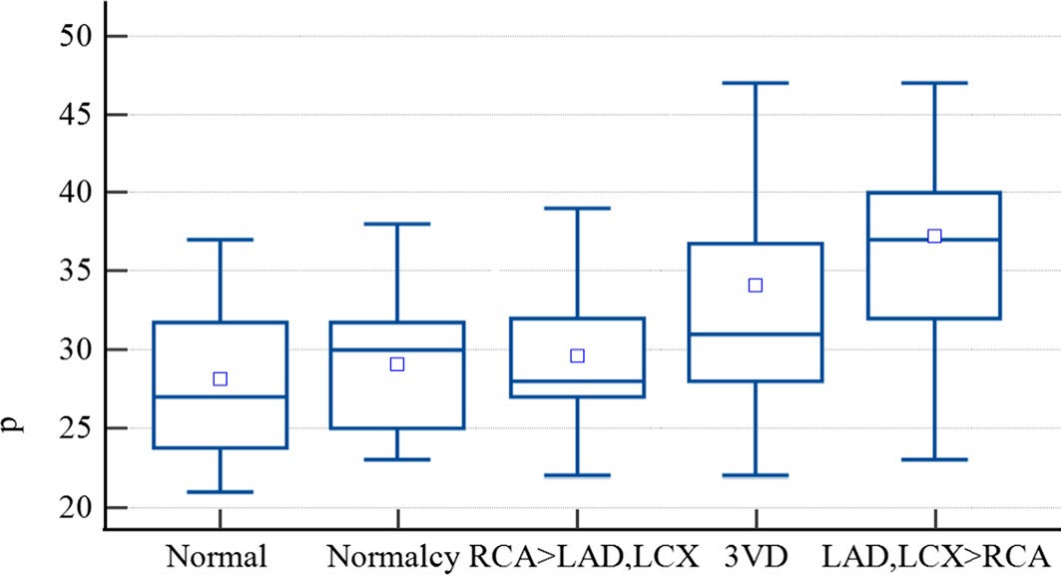
Quantitative analysis of the stress maximal RV/LV uptake ratio. Box-and-Whisker plots for each subgroup of patients are represented

Using QCard-NM, the patients in the subgroups of the SPECT/Angio dataset and the normalcy dataset were analyzed to confirm the relationship between the stress maximal RV/LV uptake ratio (within the lateral segment of the RV wall) and CAD. The results are visualized in box-and-whisker plots (see Fig. 11). The median and IQR of the maximal RV/LV in each subgroup are presented in Table 5. Post hoc test with Kruskal–Wallis was performed to test for differences between subgroup medians. It demonstrates that the maximal RV/LV uptake in the MPI scan would be significantly higher in those patients having severe LAD/LCX stenosis without severe stenosis in RCA, compared to the normal, normalcy, and RCA > LAD, LCX subgroups. Additionally, the 3VD subgroup differs significantly from the normalcy and RCA > LAD, LCX subgroups. Due to the relatively small number of individuals in the normal subgroup, no significant difference was discovered between the normal and 3VD subgroups.

**Table 5:** Quantitative values of the maximal RV/LV uptake ratio in different subgroups of SPECT/Angio and normalcy datasets

Subgroup	Median (IQR)	Post hoc—Conover
Normal	27 (24–32)	LAD, LCX > RCA
Normalcy	30 (25–32)	3VD and LAD, LCX > RCA
RCA > LAD, LCX	28 (27–32)	3VD and LAD, LCX > RCA
3VD	31 (28–37)	Normalcy and RCA > LAD, LCX
LAD, LCX > RCA	37 (32–40)	Normal, Normalcy, and RCA > LAD, LCX

Results are compared with the Kruskal–Wallis post hoc test

**Fig. 12:**
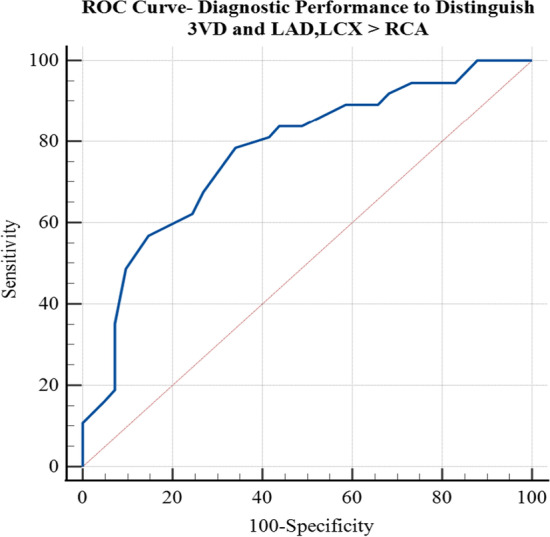
ROC curve for diagnosis of patients with severe three-vessel and/or left coronary system diseases

Moreover, a ROC curve was plotted to evaluate the performance of the stress maximal RV/LV uptake ratio for binary classification of patients belonging to 3VD and LAD, LCX > RCA subgroups from three others subgroups (see Fig. 12). The AUC was 0.772 (95% CI 0.69–0.85). This high AUC value represents that the RV/LV analysis is a good diagnostic utility for detecting patients with CAD. This outcome is comparable to the AUC for CAD detection obtained from the quantitative analysis of the LV in the MPI scan [49]. In this study, the sensitivity, specificity, and accuracy were 73.17, 67.57, and 70.51, respectively. These measures were derived using an optimal threshold equal to 30 of the stress maximal RV/LV uptake ratio within the RV wall’s lateral region. The discovered optimal threshold is also close to previously published maximal RV/LV uptake ratio threshold for detecting CAD [5].

### Visual and experimental inspection

**Fig. 13:**
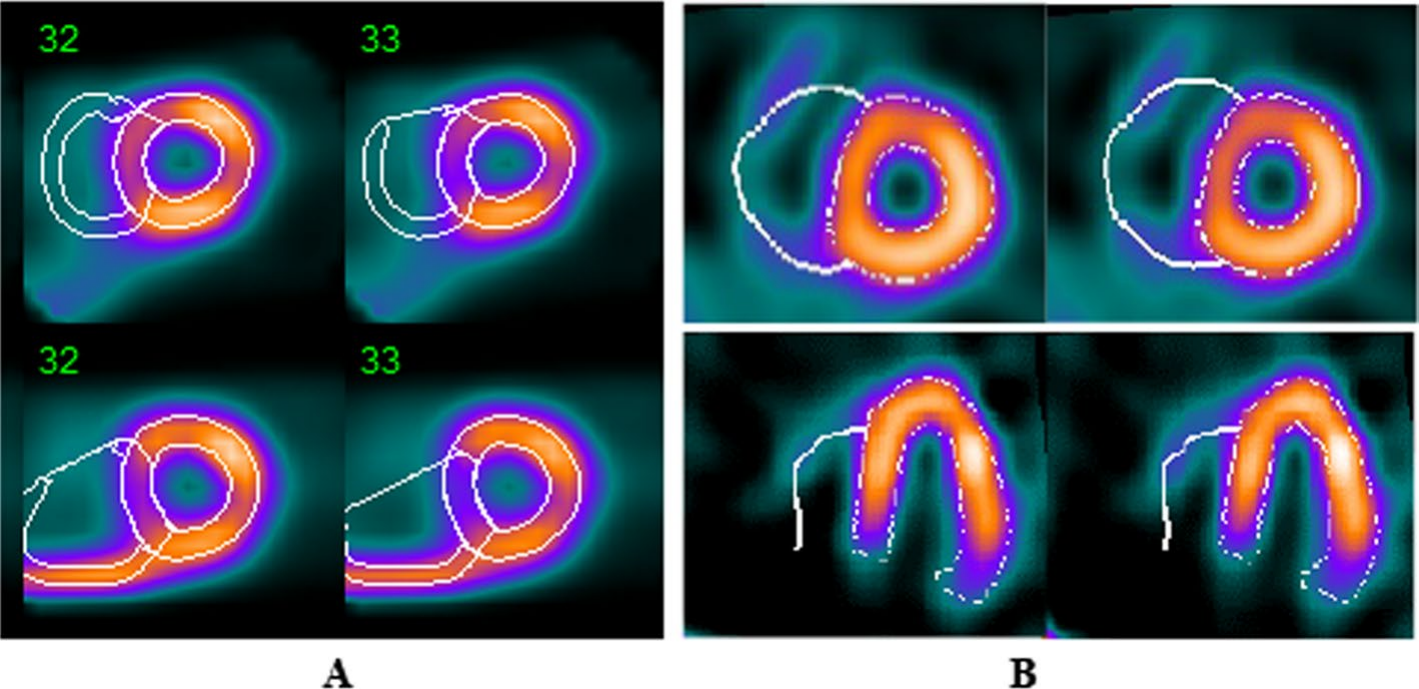
**A** QPS results in the absence (first row) and presence (second row) of extracardiac activity—**B** QCard-NM mis-segmentation in patient with high RV eccentricity index (first row); an example of the valve plane mis-localization in QCard-NM segmentation result (second row)

Figure 13 exhibits some segmentation samples where the QCard-NM and QPS algorithms failed to trace RV activity appropriately. As illustrated in Fig. 13A, extracardiac activity is the major issue in RV segmentation with the QPS. In the case of intense extracardiac activity, QCard-NM prompts the applicant to identify the RV regions from the extracardiac regions in the first step, while the iterative technique preserves the repeatability of the proposed algorithm while reducing the dependency of the final segmentation result on the applicant’s initial ROI. This approach significantly reduces the possibility of selecting an extracardiac activity as an RV region in QCard-NM.

On the other side, there are two main causes of failure in appropriate RV segmentation by QCard-NM. As Fig. 13B shows, in some MPI images, the pulmonary valve plane may be identified as misplaced by QCard-NM (see Fig. 13B). Additionally, in the case of dilated RV, the shape of the RV in non-gated MPI SPECT may be far from a spherical model. So, the proposed algorithm may fail (see Fig. 13B). However, we discovered that overall, in non-gated MPI SPECT, the spherical model is more adapted to the RV shape than the ellipsoidal or a combination of cylinder and hemisphere models. (Analysis of the latter models is not presented due to space constraints.)

As a summary, based on the results and our viewpoint, in Table 6, a brief comparison between the QCard-NM and QPS algorithms is presented.

**Table 6:** Comparison of the QCard-NM and QPS packages in RV segmentation

QCard-NM	QPS v2015.1
Spherical model	Ellipsoidal model
The segmentation results are highly dependent on the RV model	The segmentation results are highly dependent on the RV count profiles
Users can modify the model to avoid segmentation failure in the presence of extracardiac activity	Users can not interfere with the segmentation process
Automatic segmentation may fail in case of RV dilation	Automatic segmentation may fail in case of intense extracardiac uptake near the RV
Highly agreeable with the manually drawn physician’s contour	No data availability
Statistically non-significant difference between repeated supine/prone scans in RV segmentation	Statistically significant difference between repeated supine/prone scans in RV segmentation
Calculated RV cavity volume is highly correlated with actual phantom RV size	Calculated RV cavity volume is poorly correlated with actual phantom RV size
Present the quantitative RV perfusion analysis	Present the gated RV functional parameters

## Discussion

From the spatial similarity assessment section, we determined that the proposed approach is sufficiently compatible with the physician’s opinion. Due to partial volume effect in SPECT imaging, only a small portion of the RV wall exhibits significant intensity compared to the background which can be delineated with thresholding. As a result, a proper model-based approach must be used to estimate the portions of the RV wall with low signal intensity. Based on the spatial similarity result, the spherical model which is proposed in QCard-NM appears to be reasonable. However, a more complicated model, such as the one presented in [28], may produce a more accurate result, and its higher time complexity makes it unbeneficial in clinical practice.

In the repeatability assessment section, the patients were scanned in both prone and supine positions. Prone imaging results in an apparent reduction in extracardiac activity in the adjacent myocardial segment [50]. Consequently, because the main drawback of the QPS arises when there is intense extracardiac activity, the QPS approach demonstrated a larger segmented RV contour in supine images than in prone images (see Fig. 8B). However, since QCard-NM adheres strongly to the model structure, its results exhibited stable response in RV segmentation between prone and supine scans (see Fig. 8A). Furthermore, since QPS uses an ellipsoidal model as the initial model and may incorrectly track extracardiac activities, it typically estimates a higher value for the RV volume than QCard-NM (see Fig. 8C). Moreover, visual inspection of the Bland–Altman plots showed no heteroscedasticity within prone/supine segmentation. It demonstrates that the source of difference in RV segmentation between two repeated scans is independent from the RV size.

As shown in the digital phantom section, the measured RV volumes by QCard-NM have a good agreement with the actual simulated RV size. QCard-NM’s weakness is in segmenting dilated RVs. As a result, when the RV size is increased, QCard-NM estimates the RV size slightly larger than the actual size (see Fig. 10A). However, the high correlation coefficient (CC) indicates that QCard-NM is highly reliable in RV segmentation (CC: 0.88, *P* value < 0.001). In this part, the QPS algorithms performed poorly (see Fig. 10B). Since the QPS RV segmentation method has not been published, we are unable to provide a formal explanation for this observation.

In the RV/LV uptake ratio assessment section, we tried to replicate a study similar to that of Williams et al. [5].We proved that the same results could be obtained by utilizing the proposed semiautomatic segmentation and quantification algorithm. As this section shows, patients with severe CAD whose RCA has no significant stenosis can be categorized by RV quantitative analysis by utilizing QCard-NM, with an accuracy of 70.51%. An increase in the maximal RV/LV uptake ratio is likely due to two main pathophysiology reasons: (a) a global decrease in LV uptake, especially in patients with 3VD, and (b) an acute increase in RV blood flow due to stress-induced ischemic LV dysfunction [4]. We found that the sensitivity of the increased RV/LV uptake for detecting CAD is higher than its specificity. It has a low specificity since other reasons beyond coronary diseases might increase the RV/LV uptake ratio [4]. Such examples are chronic PH, exercise-induced pulmonary hypertension, acute RV strain, valvular heart disease, right ventricular hypertrophy, congenital heart disease, and chronic obstructive pulmonary disease [5, 51, 52].

Finally, based on our results and previous papers, it has been demonstrated that monitoring RV perfusion, function, and metabolism can provide valuable diagnostic and prognostic information [53, 54]; However, still, a lack of a standardized means for identifying and characterizing the “abnormal” RV in nuclear myocardial perfusion imaging exists [23, 24, 55]. The proposed QCard-NM package presents a trustworthy utility to address this shortcoming. However, there are still a lot of factors that make RV analysis challenging (as mentioned in the introduction). Investigating a variety of intervening factors is essential to identify the ideal thresholds for perfusion and functional parameters. Therefore, further studies may help to conduct a reliable quantitative analysis software package.

## Limitations

In the spatial similarity assessment section, the physician delineated the RV contours just in patients whose RV signal intensity was sufficiently high. When the RV signal intensity is high, an automatic segmentation algorithm performs reasonably well, as would be expected. Thus, further analysis is suggested to assess the precision of segmentation by independent gold standard scans such as MRI. Additionally, there is always the possibility of human error in ROI selection.

Our dataset was limited to ^99m^TC-sestamibi studies, whereas other radiotracers may have different in vivo activity distributions. Consequently, more evaluation with various SPECT radiotracers is recommended. Assessing the performance of the different RV segmentation algorithms for PET radiotracers is also recommended for comprehensive analysis.

In the digital phantom study section, we found that the shape of the RV in the averaged phantom of the 4D XCAT program was fairly congruent with the spherical model. However, it may vary in the real patient population. Therefore, it is again recommended as a follow-up study to evaluate the precision of the RV volume measured using the proposed algorithm by gold standard modalities such as MRI on a large patient population.

In the RV/LV uptake ratio assessment section, as with any retrospective study, referral bias is possible. The clinical characteristics of the patients were heterogeneous as well. Additionally, the dataset was restricted to 20 patients with low risk of CAD and 60 patients with coronary angiography; these patients were not divided into equal groups based on the coronary arteries involved. If the results of this section are going to be considered generally, these limitations should be taken into account. This concern can be lessened by a comprehensive study considering a large angiographic population with a wide range of coronary anatomic severity levels.

As mentioned in the introduction, the RV measured counts may be impacted by image acquisition and reconstruction techniques. In this study, the evaluation of RV segmentation performance in MPI SPECT has been restricted to only certain acquisition and reconstruction method. Further, it is recommended that the effects of different acquisition protocols, various scanner physical characteristics, alternative reconstruction techniques, and different correction approaches be investigated in a comprehensive study using patients’ data and simulated phantoms.

In this study, the proposed RV segmentation technique is only suitable for non-gated MPI SPECT. However, RV functional analysis has proven to be effective in diagnosing several diseases [18, 19]. Therefore, delineating the RV position and shape in Gated-SPECT may be valuable. Nevertheless, the proposed model should be modified for gated scans, whereas in the diastolic phase, a simple spherical model may not be appropriate. As a practical matter, we recommend continuing the segmentation improvements so that quantitative functional analysis of the RV in MPI Gated-SPECT with a more flexible model can be done.

## Conclusion

In conclusion, we have suggested an accurate, consistent, and efficient semiautomatic segmentation method to detect the RV in non-gated MPI SPECT images. The segmentation and quantification of the RV in the QCard-NM algorithm are based on iteratively fitting a spherical model to the RV mid-myocardium. This study has demonstrated that the RV quantification analysis by a semiautomatic software package can be beneficial in detection of those patients with 3VD or left coronary system significant stenosis.

### New knowledge gained

To quantify and segment the RV in non-gated MPI SPECT, a model-based semiautomatic segmentation algorithm has been presented. Results indicate that the RV segmentation and quantification may aid in identifying those patients with CAD (with no significant RCA stenosis).

## Supplementary Information

**Additional file 1**.** Flowchart S.1**: LV segmentation algorithm.** Flowchart S.2**: RV segmentation algorithm.** Table S.1**: Individual results of repeated scans of the supine/prone dataset.

## Data Availability

The datasets used and analyzed during the current study are available from the corresponding author upon reasonable request.

## Abbreviations

3VD: Three-vessel disease
AUC: Area under the ROC curve
CA: Coronary angiography
CAD: Coronary artery disease
CC: Correlation coefficient
CI: Confidence interval
CR: Coefficient of repeatability
DSC: Dice similarity coefficient
IQR: Interquartile range
LAD: Left anterior descending
LCX: Left circumflex artery
LoA: Limit of agreement
LV: Left ventricle
MAPE: Mean absolute percentage error
MPI: Myocardial perfusion imaging
XCAT: Non-uniform rational B-spline (NURB) extended cardiac-torso
RCA: Right coronary artery
ROC: Receiver operating characteristic
RV: Right ventricle
SPECT: Single-photon emission computed tomography
