# Supplementary material for: QCard-NM: Developing a semiautomatic segmentation method for quantitative analysis of the right ventricle in non-gated myocardial perfusion SPECT imaging
Source: EJNMMI Phys. 2023 Mar 23;10:21. doi: 10.1186/s40658-023-00539-6 (PMC10036722; doi:10.1186/s40658-023-00539-6)
Supplement: Supplementary file 1 — Additional file 1. Flowchart S.1: LV segmentation algorithm. Flowchart S.2: RV segmentation algorithm. Table S.1: Individual results of repeated scans of the supine/prone dataset. [file 40658_2023_539_MOESM1_ESM.docx]

**Supplemental Material**

**QCard-NM: Quantitative Analysis of the Right Ventricle in Non-Gated Myocardial Perfusion SPECT Imaging**

*European Journal of Nuclear Medicine and Molecular Imaging - Physics*


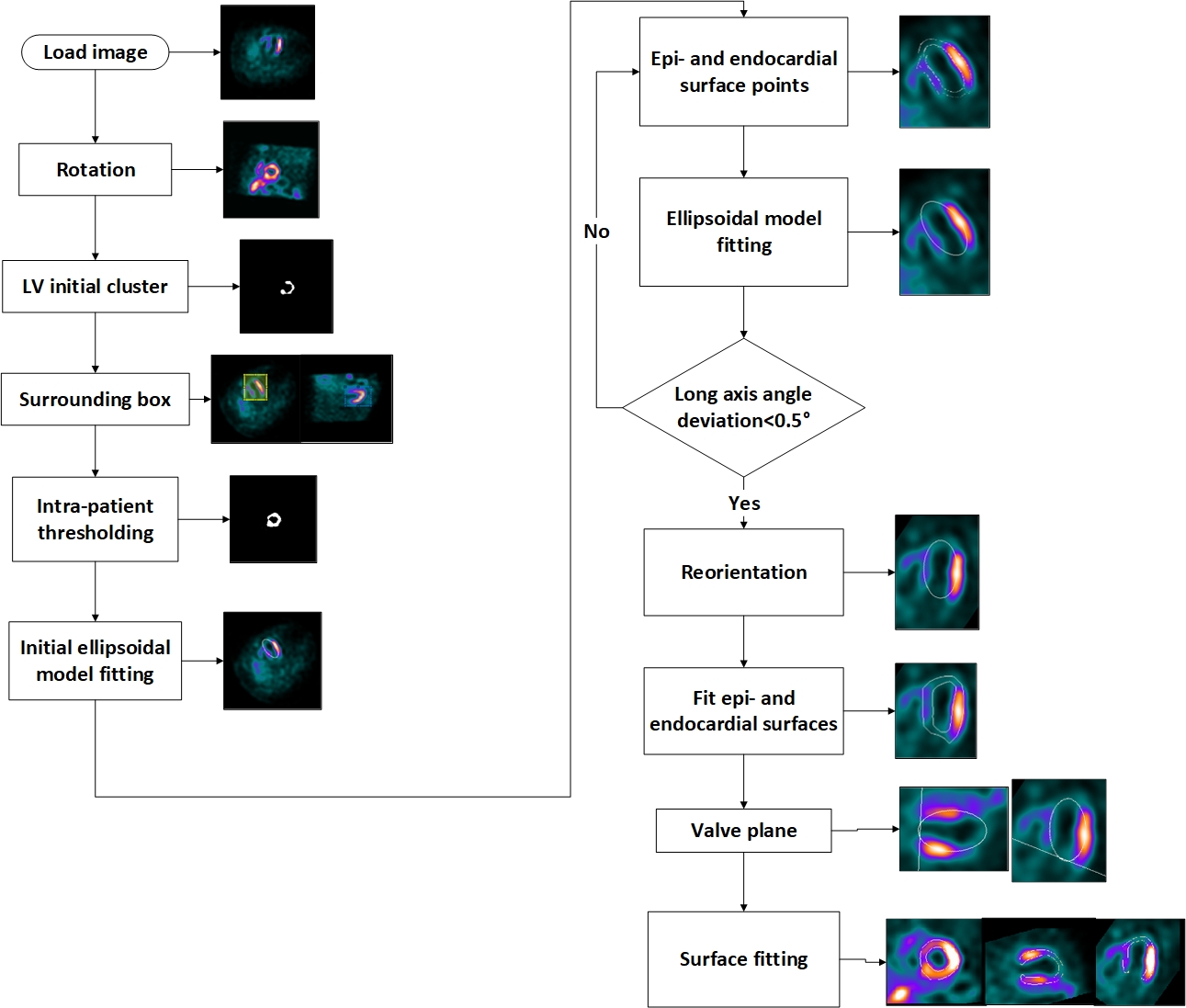


Flowchart S.1- LV segmentation algorithm


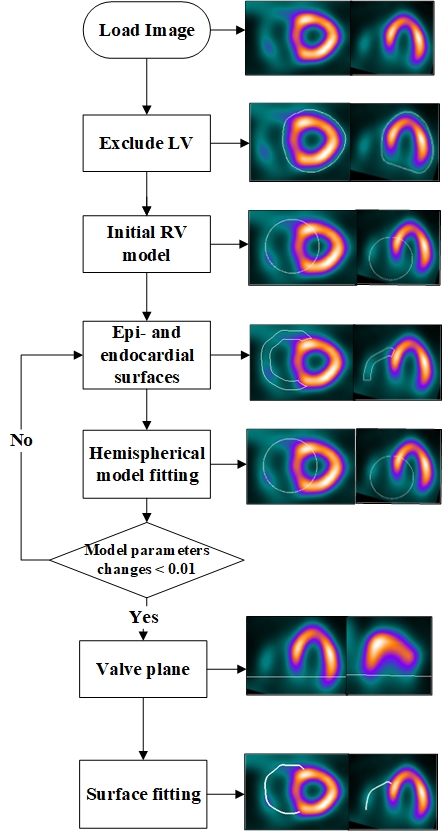


Flowchart S.2- RV segmentation algorithm

Table S.1- Individual results of repeated scans of the supine/prone dataset

| Patient Number | LV Volume (ml) - Supine Scan (Non-gated) | | LV Volume (ml) - Prone Scan (Non-gated) | | RV Volume (ml) – Supine Scan (Non-gated) | | RV Volume (ml) - Prone Scan (Non-gated) | | Maximal RV-to-LV uptake ratio - Supine (Quantitative Analysis) |
| --- | --- | --- | --- | --- | --- | --- | --- | --- | --- |
|  | QCard-NM | QPS | QCard-NM | QPS | QCard-NM | QPS | QCard-NM | QPS | QCard-NM |
| 1 | 63 | 68 | 71 | 69 | 58 | 105 | 57 | 89 | 35 |
| 2 | 31 | 32 | 32 | 30 | 27 | 39 | 29 | 27 | 25.5 |
| 3 | 23 | 22 | 34 | 24 | 19 | 36 | 24 | 25 | 27 |
| 4 | 97 | 88 | 99 | 109 | 88 | 85 | 86 | 147 | 43 |
| 5 | 36 | 38 | 32 | 40 | 26 | 61 | 27 | 32 | 30 |
| 6 | 53 | 66 | 53 | 71 | 41 | 53 | 33 | 58 | 38.5 |
| 7 | 124 | 144 | 126 | 138 | 63 | 108 | 68 | 125 | 48.5 |
| 8 | 43 | 53 | 45 | 46 | 41 | 64 | 40 | 42 | 28 |
| 9 | 39 | 39 | 37 | 40 | 48 | 58 | 35 | 24 | 33 |
| 10 | 54 | 54 | 65 | 62 | 47 | 77 | 34 | 63 | 33 |
| 11 | 111 | 102 | 98 | 111 | 104 | 102 | 97 | 102 | 30.5 |
| 12 | 81 | 76 | 72 | 73 | 76 | 118 | 60 | 93 | 35.5 |
| 13 | 64 | 75 | 84 | 94 | 38 | 39 | 42 | 40 | 44.5 |
| 14 | 51 | 51 | 47 | 50 | 53 | 43 | 46 | 32 | 33 |
| 15 | 112 | 130 | 130 | 140 | 67 | 80 | 68 | 81 | 48.5 |
| 16 | 72 | 65 | 66 | 69 | 81 | 116 | 82 | 103 | 36.5 |
| 17 | 75 | 77 | 71 | 72 | 43 | 31 | 38 | 32 | 35.5 |
| 18 | 54 | 57 | 51 | 57 | 53 | 77 | 48 | 42 | 41 |
| 19 | 76 | 65 | 63 | 63 | 41 | 44 | 48 | 27 | 35.5 |
| 20 | 55 | 63 | 54 | 71 | 41 | 56 | 36 | 51 | 33 |
| Mean ± SD | 65.65 ±  28.12 | 68.25 ±  30.26 | 66.65 ±  28.84 | 71.45 ±  32.49 | 52.94 ±  21.67 | 69.6 ±  28.36 | 50.04 ±  20.86 | 61.75 ±  36.93 | 35.75 ±  6.41 |

EDV: End Diastolic Volume; ESV: End Systolic Volume; EF: Ejection Fraction; LV: Left Ventricular; RV: Right Ventricular; SD: Standard Deviation.
